# Single cone and bioceramic sealer in oval canals: Efficiency endodontic obturation

**DOI:** 10.4317/jced.62633

**Published:** 2025-04-01

**Authors:** Nicolás Collado-Castellanos, Pedro Micó-Muñoz, Alberto Albero-Monteagudo, Annayancy Castañeda-Argueta, Mahnoor Aamir, Vicente Faus-Llácer

**Affiliations:** 1 Universidad Europea de Valencia. Faculty of Health Science. Department of Odontology; 2 London, United Kingdom; 3 Universitat de València. Faculty of Medicine and Odontology. Deparment of Stomatology. Deparment of Dental Pathology, Conservative Dentistry and Endodontic

## Abstract

**Background:**

To compare the volume of voids in oval canals obturated with single cone and bioceramic sealer (SCBC), single cone with lateral condensation or Guttacore by means of micro- computed tomography analysis. To measure the obturation time used for each technique.

**Material and Methods:**

65 uni-radicular teeth with an oval canal were selected. Canals were shaped with Protaper Next and divided into three groups according to the tested obturation technique. The time taken to obturate the canals was recorded. Each specimen was scanned using micro-CT at a voxel resolution of 25 µm. The volume of detected voids and volume of obturated canal was calculated in relation to the total volume. Voids were classified according to their location in the radicular thirds. 2D images obtained were used to calculate the percentage of filled area and percentage of voids. ANOVA test was used to assess significant differences in voids detected in the root thirds and obturation time. Multiple comparisons were made with the Bonferroni test. To analyze the root thirds Kruskal-Wallis was used and for pairwise comparisons Mann-Whitney with Bonferroni modification.

**Results:**

The highest percentage of void volume and void area was found in the single cone group with lateral condensation technique. These differences were significant in the apical third (*p*<0,01). SCBC was significantly faster (*p*<0.001).

**Conclusions:**

The three-obturation techniques achieved low void rates. SCBC had the least number of voids and was the fastest obturation technique. Clinical Relevance: In oval canals the quality of obturation carried out with these techniques were suitable and clinically acceptable. SCBC was the most efficient obturation technique.

** Key words:**Bioceramic sealer, Oval canals, Single cone technique, Root canal filling, Micro-CT analysis, Guttacore Obturator.

## Introduction

It is essential to have a good knowledge of the anatomical variations and root canal morphology in order to prepare and obturate the canals to achieve an appropriate sealing.

Depending on the type of teeth being treated the oval canals can have a prevalence of 50% (1). According to Vertucci’s type 1 classification, the lower incisors have a prevalence of 32.4% (2) and 55% in the union of the canals in type III classification of Vertucci (3).

There are limitations with the complete debridement of the oval canals through mechanical action. Without adequate cleaning of the main, accessory, or lateral canals there is a likelihood of failure in the endodontic treatment, often caused by the infective aetiology of peri-apical pathology (2).

To obturate the canals, a sealer and core material are required; the commonly used core material is gutta percha which serves as a vehicle or carrier for such sealer. The cement largely contributes to a good sealant for the canals. Epoxy resin sealer AH plus® (Dentsply DeTrey GmbH, Konstanz, Germany) is known as the gold standard because of its dimensional stability, penetration into the dentinal tubes and adhesion to the dentinal collagen. However, unlike calcium silicate, epoxy resin sealer is not bioactive (4). Calcium silicate cements are also used as an endodontic sealer. Some of their properties comprise high biocompatibility, an alkaline pH, hydrophilic, antibacterial effect, releasing calcium ions and formation of apatite crystals (5). The recommended obturation technique for these sealers is the single cone technique. According to the bibliography this technique causes the gutta percha cone not to adapt to the entire oval canal and in the middle and cervical third of the canal the sealer occupies large volume, being the potential cause of micro leakage and endodontic failure. Therefore, the physical properties and characteristics of a sealer are important for a successful root canal treatment. If the obturation is compromised, the prognosis of the treatment will be affected and the defective obturation could lead to a failed endodontic treatment (6).

There are a few long-term literature studies that have evaluated the success or failure of the bioceramics as a sealer for root canals. To date there are no significant differences in the success rates with canals obturated with bioceramic sealer or conventional sealers observed over 5 years (7,8).

With extensive research on thermoplastic techniques there have been findings of significant adaptation of the gutta percha and sealer to the morphology of the oval canal, confirming good coronal seal at the apical and coronal level. However, this was not with the single cone technique (9,10).

The main objective was to analyse the void spaces in obturated oval canals by micro-computed tomography with different techniques: single cone with bioceramic cement (SCBC) single cone and lateral condensation (SC+LC) and Guttacore® 

The secondary objective was to measure the obturation time taken for each technique.

The null hypothesis was that there were no significant differences in the total volume of voids neither in root thirds amongst the selected obturation techniques.

## Material and Methods

We used 65 lower incisors or single rooted lower premolars extracted for reasons not related to this study with the consent of the patient and without causing any harm to the patients’ health. The inclusion criterias were have a single oval canal and closed apex. The oval morphology of the canal was confirmed through radiographic findings of the canals in the buccal-lingual (BL) and mesial-distal (MD) direction. The teeth with two or more canals and accessory canals were excluded. To classify an oval canal, the BL diameter was between two and four times greater than the MD diameter at 5 mm from the apex.

-Chemical-mechanical preparation 

After opening, locating and obtaining patency of the canal with a size 10 K file, a working length (WL) of 1mm from the apical foramen was established with a size 15K file. The canals were instrumented with Protaper Next® files. The X1 was used in a brushing motion and the X2 and X3 with actions of brushing and pecking.

The files were mounted on the X smart iQ motor at a speed of 300rpm and 4Ncm. The canals were irrigated with 2% sodium hypochlorite for every time the file was used in the canal. The final irrigation of the canals was done with 2ml of EDTA at 17% (Canal Pro EDTA, Coltene, Langenau, Germany) for 1 minute and washed with 2mls of 2% NAOCl. The canals were dried with paper points X3 ® (Dentsply Sirona, Philadelphia, USA).

-Obturation of the canals and time recorded.

The teeth were divided into three equal groups (21 teeth per group) according to the obturation technique. A timer was used to measure the time it took to obturate each tooth which was then recorded into a Microsoft excel sheet.

Group A. Single cone with bioceramic.

The canal was obturated using a gutta-percha cone X3 (Dentsply Sirona, Philadelphia, USA) coated with bioceramic endodontic sealer, MTA-fillapex® (Angelus, Londrina, Parana, Brasil). Excess gutta percha was removed with a heat pluger, DiaPen and the FM tip (DiaDent, North Fraser Way, Burnaby, Canada), and the gutta-percha was immediately packed with the Machtou ¾ plugger (Dentsply Sirona, Philadelphia, USA).

Group B. Single cone with lateral condensation.

The canals were obturated using the master cone Protaper Next X3. The sealer used was the AH Plus®, it was place inside the canals in a pumping motion using the master gutta-percha cone until the working length. Digital spreader A and B were used to put 2% accessory gutta-percha with a diameter of 20. Excess gutta-percha was cut using the heat pluger, DiaPen and the FM tip at the level of the cement enamel junction, the gutta-percha was immediately packed with the Machtour ¾ condensor.

Group C. Guttacore 

AH plus sealer was placed using a X3 paper point 1mm less than the WL. The canals were obturated with a Guttacore 30. Guttacore was pre-heated in the Thermaprep plus 2 oven (Dentsply Sirona, Philadelphia, USA) using the manufacturers’ specified time. Guttacore was placed inside the canal applying apical pressure until the working length was reached, followed by condensing coronal gutta-percha apically with a Machtou ½ condenser. Once the gutta-percha set, excess was removed. 

-Study of the sample using micro-ct

After the canals of the teeth were obturated, they were stored in 100% humidity for 5 days to ensure the cement was completely cured.

For scanning the dental samples, a high-resolution micro-computed tomography (micro-CT) Scanner Quantum GX model (PerkinElmer, Waltham, USA) was used. The parameters used were 90kV and 88 µA, applying the mode ‘high resolution’, with a scanning time of 14 minutes per tooth and an angle of rotation of 360 degrees. A total of 803 projections were acquired per tooth with an isotropic pixel size of 25 µm and a resolution of 512x512x512 (12.8 mm FOV). The micro-CT images were automatically reconstructed using the software Auto Viewer (PerkinElmer, Waltham, USA).

To measure the volume of the material filled (sealer and gutta-percha) and analyse the voids inside the canals, a plugin was developed for Fiji/ImageJ, an open-source image-processing platform based on Java (11). The algorithm was developed to analyse the images using a preliminary intensity threshold of the filling material and the areas where there were voids. Subsequently, the 3D segment was dilated and a level-sets algorithm was initialised in each axial plane to find the optimal fit of the filling material contour and voids. It was calculated the volume that occupied the voids detected and the volume of the filled canal (gutta-percha and sealer) in relation to the total volume of the inside of the canals. The voids found were classified according to their position in the coronal, middle and apical third of the canals for each of the obturation techniques used.

**Figure 1:**
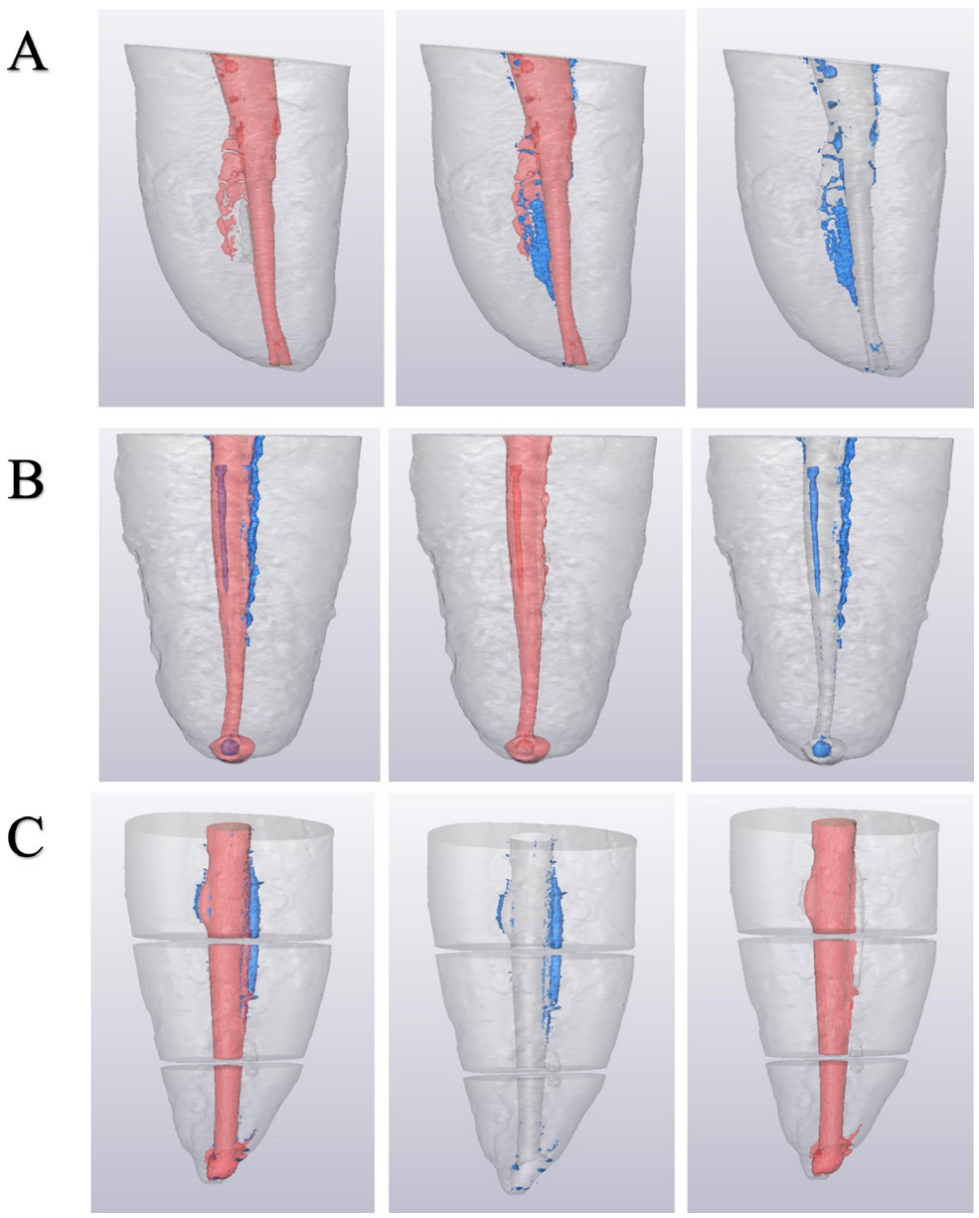
Representative micro-CT 3D reconstruction from mesial approach. Pink color represents filled material (gutta-percha and sealer), blue represents voids. Each letter corresponds to a group: A Single cone with bioceramic, B Single cone and lateral condensation, C Guttacore.

In the 2D images the area occupied by the gutta-percha and sealer and the area occupied by voids was found in order to calculate the percentage of filled area (PGFA) and the percentage of void area, (Fig. 1).

The 3D reconstruction was developed based on the generated overlays using the ImageJ software Amira 3D (Thermos Scientific, MA, USA). The 3D models differentiated the material of obturation, gutta-percha and cement (red in colour) and the voids (blue in colour) (Fig. 1).

-Statistical methodology 

To determine the size of the sample, an estimate was conducted through the F test using the ANOVA model, to reach a potency of 80%. The statistical analysis achieved a level of confidence of 95%.

A descriptive analysis of the results was carried out. The normality of the results was verified by the Shapiro-Wilk test. Parametric statistical tests were applied for the total percentage of obturation and void in the canal and non-parametric statistical tests for the root third. For Each obturation group had the ANOVA model applied. Pairwise comparisons for each group were estimated using the Bonferroni test. These methods were used to analyse the percentage of void area and the volume of voids in the canals and time taken to filled it.

Specific analysis of each root third was tested using the Kruskal-Wallis. Pairwise comparisons between groups were performed using the Mann-Whitney test with Bonferroni correction. The significance level applied in the analyses was 5% (ɑ=0.05).

## Results

-PGFA, PGVA, volume of filled and void canal.

65 samples were used, 21 teeth per group, but two teeth from group B were substituted because they experienced vertical root fractures (VRF) during lateral condensation.

Group A (SCBC) obtained an average of PGFA 94,89 ± 2,88%, the greatest percentage of the three groups, but the difference was not statistically significant (*p*>0,05) respect to groups B and C 93,29 ± 2,88%, 94,57 ± 5,40 respectively. The average of PGVA for group B (SC+LC) was of 6,70 ± 3,80%, higher than the rest of the groups, but not statistically significant ( Table 1).

SCBC and Guttacore had significantly lower percentage area of voids in the apical third in comparison to the technique of SC+LC (*P* < 0.01).

**Figure 2:**
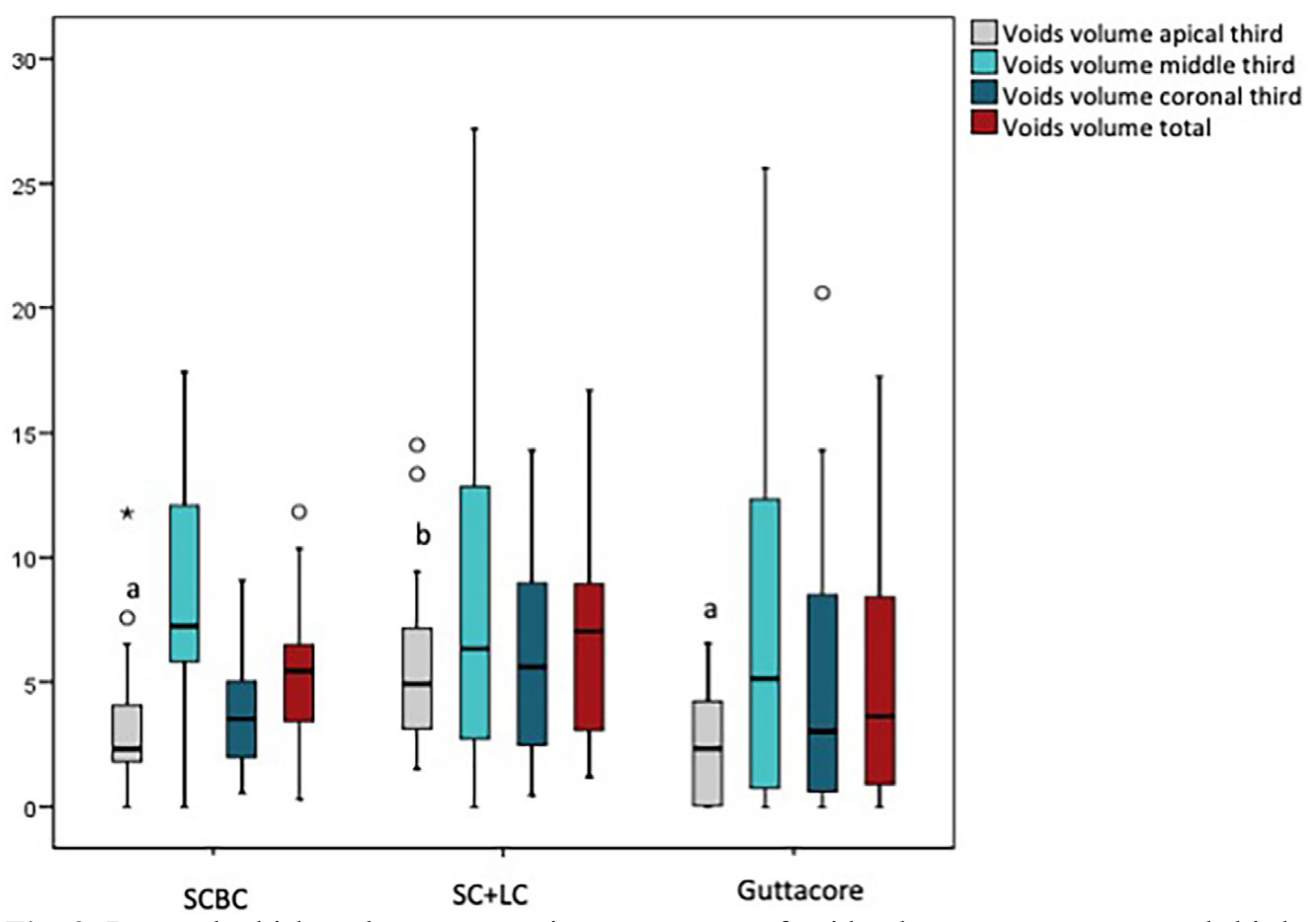
Box-and-whisker plots representing percentage of void volume among root canal thirds. The median corresponds to the horizontal line that divides the box. Significant differences were found in apical third. Different lowercase letters show significant differences among groups (*P* < 0.01).

The average filled volume for group A was of 4,086 ± 0,767 mm3 and the volume of voids were 0,220 ± 0,121 mm3, but for group B 4,518 ± 1,014mm3 y 0,319 ± 0,207mm3 and for group C 4,634 ± 1,472 mm3 y 0,257 ± 0,292mm3 respectively. Table 1 shows the measured relative percentages of the volume obturated and the volume of voids for each group and root canal third. There was no significant difference in relation to the relative mean volume (*P* = 0.429) for each group. Kruskal-Wallis test revealed significant differences (*P* < 0.01) in the percentage volume of voids in the apical third. Pairwise comparison showed group B had percentage volume of voids significantly greater than Group A (*P* < 0.005) and group C (*P* < 0.01) (Fig. 2).

Time of obturation 

**Figure 3:**
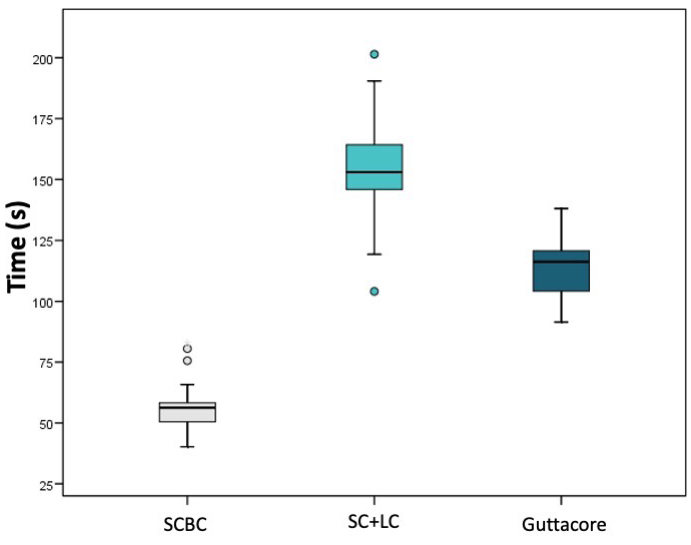
Box-and-whisker plots representing mean time taken for obturation. Different lowercase letters show significant differences among groups (*P* < 0.001).

The group that showed the least time to obturate was SCBC, 57.11 seconds, followed Guttacore, 113 seconds, and SC+LC, 152 seconds. There were significant differences between the groups (*P* < 0.001). The single cone with bioceramic technique is significantly faster than the single cone and lateral condensation and carrier-based technique (*P* < 0.001). In addition, Guttacore is significantly faster than the single cone with lateral condensation technique (*P* < 0.001) (Fig. 3).

Single cone bioceramic group demonstrated the lowest percentage of voids and was the fastest technique in obturating the oval canal followed by Guttacore group.

## Discussion

None of the three obturation techniques achieved 100% filled area or volume and have demonstrated to have a percentage area of voids within the canals, showing significant differences in the apical third. The single cone technique with lateral condensation had the highest percentage of voids, this must have been because of the limitation to adapt to the anatomy of the canal; the difference with the thermoplastic techniques such as warm vertical condensation or carrier-based technique (6,9,12). During the present work two of the 21 lower incisors fractured from group SC+LC and were subsequently replaced with two more teeth with an oval canal.

In some *in vitro* studies where oval canals or lower incisors were selected, some percentage of fractures occurred, up to 5 teeth in the same set that the present study (12,13). These authors suggested that the lateral condensation and warm vertical condensation (WVC) technique may cause more dentinal cracks that single cone technique. These cracks can cause a future VRF because of the forces and stress on the internal canals (13,14).

Li GH *et al*. (2014) used to obturate the oval canals a master cone of the same apical diameter and the same taper as the last file used (40.06) with accessory gutta-perchas. The analysis using micro-CT did not describe in detail the percentage area of voids and gaps and only showed some specimens, therefore comparison of results cannot be made. Authors used scanning electronic microscope (SEM) to observe transversal cuts. They obtained significant differences in the percentage area of voids being greater in the lateral condensation technique respect to Guttacore (15). In a similar study, SEM analysis reported an area of void significantly greater in SC+LC technique than WVC technique; warm obturation technique filled in greater quantity the oval canals (16). Warm obturation techniques can adapt the gutta-percha to irregular morphology of the root canal as it flowed with the cement and adapted to the (6,16). Both, carrier-based technique and WVC can obturate the canal up to 90% and reduce the percentage of voids (17,18) according to the present study.

Group A, single cone and bioceramics, showed a percentage voids area of 5,11 ± 2,88%. This corresponds to the gaps or failure between the sealer and the gutta-percha or the sealer and dentine walls. In the apical zone resulted in the least percentage, 3,2%, it was not significantly different to the thermoplastic technique but was significantly different from the SU +LC technique.

Celikten *et al*. (2016) found a lower percentage of voids in obturated oval canals with the single cone and bioceramics technique. These were compared with the single cone technique and epoxy resin sealer without significant differences found (19). Similar results in percentage area of gutta percha, sealer and voids were reported, the percentage of voids was found to be somewhat higher, than that observed in the present study, 4,28% vs 3,2% in the apical region (18).

In the volumetric analysis, the technique of SCBC and the technique of Guttacore showed the least percentage volume of voids, being significantly less in the apical third with respect to the SC+LC technique. In contrast to Keles *et al*. (2014), they found a percentage volume of voids significantly higher in the middle and coronal thirds using the technique of lateral condensation compared to the technique of WVC (20).

As we observed in the PGFA, warm gutta-percha techniques could generate less voids. Li GH *et al*. (2014) found significant differences in the percentage void volume between the Guttacore and lateral condensation in the three thirds of the root canal (15). Comparing SC+LC technique versus WVC technique, SC+LC technique reporting poorer fit of the gutta-percha and sealer to the canal walls. The differences in the percentage void volume distribution were significant in the apical and coronal third, similar results to the current study. An experimental non-guttapercha-based (NGP) filling material was used to obturated oval canals resulting in a higher percentage of void volume in comparison with SC+LC (16). NGP was combined with a hybrid sealer, composed by epoxy resin and methacrylate and lateral condensation.

The obturation with SCBC Endosequence HiFlow® demonstrated a lower percentage volume of voids, filling the canal in 96-99% of total volume of the canal, like the current experimental study. Nevertheless, WVC with Endosequence Hiflow seemed to fill better the oval canals, because less volume of voids was reported being the differences significant in the overall average as in the coronal third. It is interesting to find that the percentage volume of voids in 6 months increased in the SCBC HiFlow group indicating an increase in the porosity of the sealer therefore the appearance of large number of voids (21).

Single cone and lateral condensation techniques used in oval canals are moving away from the rules of obturation according to Wu y Cols 2000 because in the coronal and middle third of the canals are a large quantity of sealer filling the vestibular and lingual extensions. It could cause bacterial microleakage due to the contraction and solubility of the sealing cement (1,18). The use of bioceramic endodontic sealer poses a change in the ideal criteria of obturation, the sealer can occupy a large volume inside the canal because of their characteristics; large amounts of sealer have not been linked to greater microfiltration or presence of gaps between the sealer and dentine because of their dimensional stability and the alkalinity. These characteristics allow the penetration of the sealer in the dentinal tubules and therefore the reduced number of gaps (22).

However, different results have been reported in the literature. A very low percentage volume of voids had been reported in oval canals filled using SC technique and different bioceramics sealers. 0,21% - 1,55% against 3,1% - 8,2% in the present study (19). In the other way, 13% and 15% volume of voids at middle and coronal third (5mm and 10mm) was observed when Endosequence BC Sealer® and single cone technique and lateral condensation technique were used (23). These differences could be caused by variation in shaping and obturation techniques and the sections made to study images, since only two sections of the canal were chosen.

The distribution of the voids could be attributed to protocols of irrigation, conservative methods, observational technique too, but their occurrence seems to be unpredictable, in line with the statements of Keleş A *et al*. (20).

The time taken to fill the canal was recorded in order to see which filling technique was faster and whether there was a relationship between time and filling capacity. The single cone technique with bioceramics was the fastest, together with Guttacore. In addition, these two demonstrated the least percentage of voids.

The time taken to obturate with SCBC was 57,11 ± 11,3 seconds, with Guttacore 113 ± 13,1 seconds and for SC+LC 152 ± 23,6 seconds. The technique of single cone shows a substantial time saved, especially when obturating various canals in the same session, since obturating a single canal has been carried out in less than one minute (57 seconds), nearly 3 times less than the single cone and lateral condensation technique and half as much as the carrier-based technique. Similar results were observed when SC+LC was compared to Hybrid technique (gutta-percha cone and Thermafil). The carrier-based technique required significantly less time of work and had uniform gutta-percha distribution to the root surface, favouring a three-dimensional obturation with minimum gaps (24).

Previous study showed that single cone and epoxy resin sealer in curved canals also resulted be much faster (44 ± 4,32 seg) (25). A common agreement amongst the authors was that the vertical and lateral condensation technique were the slowest comparing with other techniques, as time taken varies between 181 seconds and 310 seconds (26).

The null hypothesis was partially rejected because there was significant difference in volume of voids in the apical third.

## Conclusions

The three obturation techniques used to obturate oval canals: single cone with bioceramics, single cone with lateral condensation and Guttacore filled the canal in large extent and none of them were free of voids. But the single cone with bioceramics technique was the most efficient, measured in terms of fewer voids remaining and faster. This means less treatment time for the patient and more clinical productivity.

## Figures and Tables

**Table 1:** Mean values and standard deviation (percentage) of the filled canal volume, void volume, filled area and void area by obturation technique and root canal third. Different superscript letter indicates significant difference among the groups (*P* < 0.001).

Regions	Groups	Mean Filled Area (%)	Mean Void Area (%)	Mean Filled Volume (%)	Mean Void Volume (%)
Coronal third	SCBC	96.30 ± 2.29	3.70 ± 2.29	96.32 ± 2.28	3.68 ± 2.88
	SC+LC	93.54 ± 4.25	6.50 ± 4.28	93.76 ± 3.98	6.24 ± 3.98
	Guttacore	94.78 ± 5.76	5.21 ± 5.76	94.82 ± 5.80	5.18 ± 5.80
Middle third	SCBC	91.71 ± 5.29	8.29 ± 5.29	91.76 ± 5.35	8.24 ± 5.35
	SC+LC	91.29 ± 6.31	8.68 ± 6.31	91.60 ± 6.69	8.40 ± 6.69
	Guttacore	92.91 ± 7.27	7.09 ± 7.27	92.92 ± 7.28	7.08 ± 7.28
Apical third	SCBC	96.79 ± 2.76	3.21 ± 2.75 ^a^	96.94 ± 2.82	3.06 ± 2.82 ^a^
	SC+LC	94.20 ± 3.60	5.79 ± 3.59 ^b^	94.20 ± 3.60	5.80 ± 3.60 ^b^
	Guttacore	97.49 ± 2.24	2.50 ± 2.24 ^a^	97.44 ± 2.20	2.56 ± 2.20 ^a^
Total	SCBC	94.89 ± 2.88	5.11 ± 2.88	94.89 ± 2.88	5.11 ± 2.88
	SC+LC	93.29 ± 3.80	6.70 ± 3.80	93.30 ± 3.80	6.70 ± 3.80
	Guttacore	94.57 ± 5.40	5.43 ± 5.40	94.58 ± 5.41	5.42 ± 5.41

## Data Availability

The datasets used and/or analyzed during the current study are available from the corresponding author.
